# Genetic forms of tauopathies: inherited causes and implications of Alzheimer’s disease-like TAU pathology in primary and secondary tauopathies

**DOI:** 10.1007/s00415-024-12314-3

**Published:** 2024-03-30

**Authors:** Felix Langerscheidt, Tamara Wied, Mohamed Aghyad Al Kabbani, Thilo van Eimeren, Gilbert Wunderlich, Hans Zempel

**Affiliations:** 1 Institute of Human Genetics, Faculty of Medicine and University Hospital Cologne, University of Cologne, 50931 Cologne, Germany; 2 Center for Molecular Medicine Cologne (CMMC), University of Cologne, 50931 Cologne, Germany; 3 Department of Natural Sciences, Bonn-Rhein-Sieg University of Applied Sciences, Von-Liebig-Str. 20, 53359 Rheinbach, Germany; 4 Multimodal Neuroimaging Group, Department of Nuclear Medicine, Faculty of Medicine and University Hospital Cologne, University of Cologne, 50937 Cologne, Germany; 5 Department of Neurology, Faculty of Medicine and University Hospital Cologne, University of Cologne, 50937 Cologne, Germany; 6 Center for Rare Diseases, Faculty of Medicine and University Hospital Cologne, University of Cologne, 50931 Cologne, Germany

**Keywords:** Genetic tauopathy, *MAPT*, TAU, Primary tauopathy, Secondary tauopathy, Alzheimer’s disease

## Abstract

Tauopathies are a heterogeneous group of neurologic diseases characterized by pathological axodendritic distribution, ectopic expression, and/or phosphorylation and aggregation of the microtubule-associated protein TAU, encoded by the gene *MAPT*. Neuronal dysfunction, dementia, and neurodegeneration are common features of these often detrimental diseases. A neurodegenerative disease is considered a primary tauopathy when *MAPT* mutations/haplotypes are its primary cause and/or TAU is the main pathological feature. In case TAU pathology is observed but superimposed by another pathological hallmark, the condition is classified as a secondary tauopathy. In some tauopathies (e.g. *MAPT*-associated frontotemporal dementia (FTD), progressive supranuclear palsy (PSP), corticobasal degeneration (CBD), and Alzheimer's disease (AD)) TAU is recognized as a significant pathogenic driver of the disease. In many secondary tauopathies, including Parkinson's disease (PD) and Huntington's disease (HD), TAU is suggested to contribute to the development of dementia, but in others (e.g. Niemann-Pick disease (NPC)) TAU may only be a bystander. The genetic and pathological mechanisms underlying TAU pathology are often not fully understood. In this review, the genetic predispositions and variants associated with both primary and secondary tauopathies are examined in detail, assessing evidence for the role of TAU in these conditions. We highlight less common genetic forms of tauopathies to increase awareness for these disorders and the involvement of TAU in their pathology. This approach not only contributes to a deeper understanding of these conditions but may also lay the groundwork for potential TAU-based therapeutic interventions for various tauopathies.

## Introduction

Tauopathies are a group of clinically, morphologically, and biochemically heterogeneous neurodegenerative diseases characterized by cognitive decline and dementia [1, 2]. They share the common neuropathological characteristic of deposits or ectopic presence of the microtubule-associated protein TAU in the brain [3]. TAU is encoded by the *MAPT* (microtubule associated protein TAU) gene on chromosome 17q21, there are six different isoforms of TAU expressed in the human brain as a result from alternative splicing of exons 2, 3, and 10 [4, 5]. Human TAU protein comprises four areas: an N-terminal projection domain, a proline-rich domain, a microtubule-binding domain (MBD), and a C-terminal domain. These can form either three isoforms, each with four microtubule-binding repeats, referred to as 4R TAU, or three isoforms lacking the second repeat, referred to as 3R TAU. Another high-molecular weight-isoform of TAU (“big TAU”) is mainly expressed in the human peripheral nervous system, but its pathological relevance is underexplored [6, 7]. Under physiological conditions, TAU is predominately present in the axon of neurons [8]. In tauopathies, TAU is hyperphosphorylated and accumulates in the soma, eventually aggregating as neurofibrillary tangles (NFTs) [1, 9].

Primary tauopathies are diseases where either the presence of TAU filaments or ectopic presence of (phosphorylated) TAU is the main or sole known abnormality, or where TAU pathology is the major driver of neurodegeneration [10]. The spectrum of these primary tauopathies comprise e.g. *MAPT-*associated-Frontotemporal Dementia (FTD), Pick’s disease (PiD), Progressive Supranuclear Palsy (PSP), and Corticobasal Degeneration (CBD). *MAPT* mutations associated with neurodegenerative diseases may play a role in enhancing TAU aggregation, disruption of TAU protein structure, and/or interfering with mRNA splicing of *MAPT* [11, 12]. Approximately 30% of primary tauopathy cases were reported with family history of dementia or primary tauopathy [13, 14].

In contrast, in secondary tauopathies TAU pathology likely develops in response to other pathogenic events. Here, no pathogenic mutation can be found in *MAPT* itself, TAU is not considered the primary pathogenic cause, or TAU’s exact contribution to disease progression is unclear [2, 12]. In Alzheimer’s disease (AD), the most common form of dementia, both Amyloid-β (Aβ) and TAU drive the pathology, as oligomeric Aβ as well as TAU mislocalization, phosphorylation and aggregation can also be faithfully modelled in mice and human neurons [15–17]. Examples of other secondary tauopathies include Huntington’s disease (HD), Lewy body dementia (LBD), Parkinson's disease (PD), Niemann-Pick disease type C (NPC), Down syndrome (DS), and myotonic dystrophy (DM) [12]. For many secondary tauopathies, the (genetic) trigger is well established, but the disease mechanism finally leading to TAU pathology and neuronal dysfunction is often unclear. As genetic risk factors and the corresponding pathomechanisms may contribute to TAU-related dementias, understanding genetic and rare tauopathies may be the key for elucidating the pathogenic cascades also of more common tauopathies such as AD. In the following sections, we will briefly introduce primary and secondary tauopathies with a focus on genetic diseases and discuss mechanisms of action.

## Primary tauopathies

Primary tauopathies comprise diverse neurologic disorders classified as Frontotemporal Lobar Degeneration (FTLD) with TAU-pathology, referred to as FTLD-TAU [1, 18, 19]. These disorders are characterized by the abnormal aggregation and/or accumulation of TAU protein primarily within neurons or glial cells [20, 21]. The specific type of tauopathy determines the affected cell types and contributes to the variability of clinical symptoms [22–25]. Most commonly, these conditions lead to clinical dementia syndromes, typically with an onset before the age of 65 [19, 26]. Primary tauopathies can be categorized using various criteria, such as their sporadic or familial nature based on mutations in the *MAPT* gene, or by the predominant TAU isoforms involved, resulting in 3R, 4R, or 3R/4R tauopathies [1, 18, 21].

Up to 30% of primary tauopathy cases appear to be familial, while the majority are sporadic [13, 14, 20]. In familial cases, an autosomal dominant inheritance pattern is predominant, with mutations mainly identified in three genes (*C9orf72*, *GRN*, or *MAPT*). Each of these genes contributes causatively to familial cases, with roughly equal prevalence, accounting for 5–10% each [13, 27, 28]. In most cases, there is no convincing evidence for differences between different populations/ethnic groups with respect to specific mutations causing neurodegenerative primary and secondary tauopathies [29], but differences in sporadic forms of tauopathies were described, in particular with respect to biomarkers for AD and mild cognitive decline [30]. The genetic profiles of some tauopathies, i.e. FTD, PSP, CBD, PD, might show differences across ethnic groups, since the *MAPT* gene has two haplotypes (H1 and H2). H1 haplotypes are found in all ethnic groups and are associated with various neurodegenerative disorders. On the other hand, H2 haplotypes are primarily found in Europeans and southwest Asians and are linked to a reduced risk of developing neurodegenerative disorders [31].

The following section provides an overview of selected primary tauopathies and their (potential) genetic causes.

### *MAPT*-related frontotemporal dementia with Parkinsonism-linked to chromosome 17 (*MAPT*-related FTD/FTDP-17)

Over 50 mutations have been discovered so far in the *MAPT* gene, most of which lead to the manifestation of the *MAPT*-related FTD phenotype. This neurodegenerative disorder is also classified among the 4R primary tauopathies (although in a few isolated cases, 3R TAU was predominant), and is characterized by a clear genetic origin: The disease-causing mutations, including missense and splicing variants, affect exonic and intronic regions of *MAPT* [32, 33]. Missense mutations often impact TAU's interaction with microtubules, leading to enhanced TAU aggregation. Such mutations are commonly localized in the microtubule binding domain, while others in the C-terminus disrupt TAU's ability to bind to microtubules, influencing axonal transport or microtubule assembly [34, 35]. Splicing mutations in intron 10 shift the isoform ratio towards 4R, resulting in its overproduction and assembly in TAU filaments [36, 37].

The type and location of the mutations determine whether *MAPT*-associated FTD results from a loss of function (LoF) or a toxic gain of function (GoF) by the assembly of TAU filaments [37–40]. The presence of filamentous TAU deposition serves as a hallmark in all tauopathies. Notably, in *Mapt*-KO mice, minimal phenotypic manifestations are evident, with only mild behavioral anomalies, such as hyperexcitability, and subtle cellular-level impairments like compromised long-term depression [34, 35]. A toxic GoF may occur when an excessive amount of TAU resulting from elevated 4R ratios overwhelms the available binding sites on microtubules, thereby resulting in the assembly of unbound TAU in filaments [41, 42].

TAU is a natural therapeutic target for *MAPT*-related FTD, and an attractive target for related tauopathies such as AD, as there is a strong correlation between the amount of NFTs and cognitive decline, and *Mapt*-KO mice are healthy. So far, however, TAU-based therapies have not shown clinical efficacy [43]. Currently pursued strategies include decreasing total levels of TAU via immunological removal, genetic suppression via antisense oligomers (which are also used for splicing modulation) or microRNAs, inhibition of posttranslational modifications, and more recently also specifically targeting TAU interaction motifs or expressing proteins inducing TAU degradation (reviewed e.g. in Self and Holtzman [44], for a new TAU interaction motif inhibitor see Roth et al. [45] and for TRIM11-mediated TAU reduction see Zhang et al. [46]). In particular for AD there is considerable academic and industrial interest in developing TAU-based therapies, which may also be helpful in other (genetic) tauopathies, in particular for those where TAU is the main disease driver, e.g. PSP, CBD, AGD and PiD as listed below.

In conclusion, the diverse mutations within the *MAPT* gene associated with disease reveal a complex genetic basis for *MAPT*-related FTD, with some mutations potentially leading to a loss of TAU’s canonical MT-binding function, and others causing a toxic GoF, both resulting in TAU filament assembly.

### Progressive supranuclear palsy (PSP)

PSP is a mainly sporadic 4R tauopathy, with familial cases making up less than 5% of affected patients. Genetic variations (such as the H1 and H2 haplotype, which will be discussed in further detail below) within the *MAPT* gene were suggested to confer increased risk for PSP [47, 48]. Although more than ten other risk loci, including genes like *STX* or *MOBP* (for a complete list, see Table 5 in Wen et al. [49]), have been identified for PSP, the importance of *MAPT* mutations remains central, with 15 such mutations identified in PSP patients. Patients with *MAPT* mutations face an earlier onset of disease compared to those linked e.g. to *LRRK2* or *DCTN1* mutations [14, 49].

PSP affects both gray and white matter, with TAU inclusions found in specific brain regions, including 4R TAU-based neurofibrillary tangles and globular inclusions, which are histologically difficult to distinguish from Pick bodies [50]. Distinctive features such as TAU-positive tufted astrocytes and coiled bodies were also observed [51] (for review see Götz et al. [1]).

The clinical presentation of PSP exhibits numerous similarities with Parkinson's disease, making the specific diagnosis of PSP challenging. Common motor impairments, such as impaired balance and spontaneous falls, are frequently observed and occur early. Additionally, cognitive changes and a variety of other symptoms have been documented. Unlike Parkinson's disease, tremor is not a characteristic feature of PSP. In contrast, PSP patients often display unique symptoms like vertical gaze palsy, which are not typically observed in Parkinson's patients [52].

Epigenetic factors like methylation impact *MAPT* expression in PSP patients. Notably, CpG1 hypomethylation in *MAPT* intron 0 was found in frontal cortices of PSP patient brains, increasing *MAPT* mRNA. Furthermore, a genome-wide methylation study revealed a cluster of differential methylation probes in the chromosomal region chr.17q21.31, which includes *MAPT*, the major risk gene for PSP [53] (for review see Debnath et al. [54]). In addition to *MAPT*, specific single nucleotide polymorphisms (SNPs) have been implicated in PSP pathology, contributing to prolonged disease duration and subcortical pathology. These SNPs are associated with the *TRIM11* gene and intron 3 of *SLC2A13* which is in close proximity of *LRRK2*. *LRRK2* is associated with an enhanced survival rate in PSP [55]. Notably, case–control GWAS studies highlight *MAPT* as the key risk locus for PSP with the strongest effect size [54, 56, 57].

A main feature of PSP in most patients is L-DOPA non-responsive parkinsonism (referred to as PSP-P) [26, 58]. Richardson syndrome (PSP-RS) shows an independent spectrum of symptoms including postural instability and subcortical dementia [59].

A recent development in the realm of PSP is the emergence of a subtype defined as protracted course PSP (PC-PSP), characterized by a longer disease duration, slower clinical progression and anatomically restricted neuropathological symptoms [59]. Further research is needed to understand this syndrome in more detail, as only a limited number of cases have been identified and studied thus far, which is not yet sufficient for genotype–phenotype studies.

### Corticobasal degeneration (CBD)

CBD, categorized as a sporadic 4R primary tauopathy, shares significant neuropathological and etiological parallels with PSP [20, 26]. Clinically, more than 50% of CBD cases initially present with apraxia in one arm, often accompanied by a gait disorder affecting motor function. Behavioral changes and speech disorders were also reported, as well as symptoms such as ideomotor apraxia, dementia, clumsiness of the limbs, early tremors, and the alien-limb phenomenon. Progressive development includes asymmetric, unilateral parkinsonism, cognitive decline, and gait impairment. Additional features in CBD patients consist of myoclonus and sensory disturbance. Typically, at least one parkinsonism symptom is evident in CBD [60].

Similar to the observations in PSP, CBD was linked to a limited number of identified *MAPT* mutations (most commonly p.G389R and p.N410H) as causative factors [26, 61]. In CBD patients, TAU-positive structures are more widely distributed, impacting both white and gray matter [62, 63]. CBD primarily affects the cerebral cortex and basal ganglia, and, similar to accumulations of 4R TAU in PSP, these accumulations are found in neurons and glial cells [20, 64]. Unlike in PSP, NFTs are rare in CBD, while pretangles are a common hallmark in the cerebral cortex and subcortical nuclei of CBD patients. Pretangles, initially defined as non-fibrillary TAU deposits in neurons in Alzheimer's disease, represent an early stage in the pathological process of TAU protein accumulation, often considered precursors to the more advanced and fully formed NFTs, which are highly aggregated TAU protein clumps found in neurons [18, 65]. One of the most distinct pathologies in CBD is the presence of astrocytic plaques, particularly abundant in the cerebral cortex [1, 66].

To unravel CBD's genetic basis, more genotype–phenotype studies are required, complicated by its rarity and substantial pathological and etiological overlap with other tauopathies. Nevertheless, among the identified risk genes, *MAPT* is the most prevalent and important one associated with CBD [14, 67].

### Genetic factors shared in PSP and CBD

In both PSP and CBD, the overrepresentation of the H1 haplotype of Chromosome 17 (where the *MAPT* gene is located) is a significant genetic risk factor for PSP and CBD. The risk is attributed to a linkage disequilibrium caused by a 900 kb inversion of H1 that occurred 3 million years ago, resulting in two haplotypes: H1 and H2. So far, a total of five haplotypes and subhaplotypes have been identified that are significantly associated with an increased risk of PSP manifestation. These include haplotype H2, as well as the H1 subhaplotypes H1c, H1d, H1g, and H1o. Notably, H1c and H1d also contribute to CBD risk, emphasizing the shared genetic background of both disorders [48, 68, 69]. For other haplotypes (H1b, H1e H1h, H1m, H1r and H1q), no significant association with an increased risk was found [70].

The H1 haplotype elevates *MAPT* gene expression by 1.5 times compared to H2, particularly for 4R isoforms [71]. This enhances the accumulation of pathological TAU, and hence potentially also TAU toxicity. Despite being considered a risk factor, the H1 haplotype also promotes protective effects, influencing the alternative splicing of exon 10 to favor the formation of more 3R TAU isoforms [48, 69, 70, 72]. It is worth noting that H1 may be associated with a reduced regional gray matter volume in healthy carriers perhaps increasing the risk of developing sporadic cases of tauopathies such as PSP or CBD [73]. The *MAPT* H2 haplotype has a confirmed protective effect and reduces CBD risk significantly, but no *MAPT* haplotypes were directly associated with any TAU pathology measures [48].

In conclusion, the H1 haplotype on Chromosome 17, along with its subhaplotypes, represents a significant genetic risk factor for both PSP and CBD, highlighting the shared genetic background of these disorders, with complex effects on TAU pathology and isoform regulation.

### Pick’s disease (PiD)

Pick's disease (PiD) is distinguished as the sole primary 3R tauopathy (see below for secondary 3R tauopathies). Here, hyperphosphorylated filaments, exclusively composed of the 3R TAU isoforms, undergo further transformation increasing insolubility and aggregation, eventually forming structures known as Pick bodies, which represent the most distinctive hallmark of PiD [1, 74], despite rare reports of significant neuronal 4R-TAU accumulation [75]. Additionally to the Pick bodies, also cortical atrophy predominantly found in the frontal and temporal poles is a common neuropathological finding [76]. Since TAU aggregates/Pick bodies are the main and most important pathological event of PiD, it is classified as a primary tauopathy [77].

Clinical symptoms in PiD vary based on cortical atrophy location. In cases where the temporal lobe is affected, Klüver-Bucy syndrome may arise, impacting behavior with symptoms like dietary changes or visual agnosia among others. Notably, this syndrome can also be triggered by trauma to the temporal lobe [76, 78]. Conversely, pathology in the frontotemporal lobe may give rise to frontal lobe syndrome, encompassing a spectrum of symptoms ranging from behavioral changes and memory deficits to language disorders and mutism [76].

PiD is a relatively rare tauopathy, and familial cases of the disease are infrequent, with the majority of instances being sporadic. Some hereditary PiD cases are associated with missense mutations and small deletions in the *MAPT* gene, such as the G272V, ∆K281, and Q336H mutations [79–81]. The *MAPT* H2 haplotype is associated with an increased risk of PiD, while the H1b and H1f haplotypes of *MAPT* appear to be protective [82]. Isolated cases of PiD have been associated with mutations in the *PSEN1* gene [83–85].

In summary, PiD is characterized by TAU aggregates called Pick bodies and cortical atrophy in frontal and temporal poles resulting in diverse clinical symptoms. Familial PiD cases are rare, some associated with *MAPT* mutations, increased risk linked to the H2 haplotype of *MAPT,* and isolated cases linked to *PSEN1* mutations.

### Argyrophilic grain disease (AGD)

In contrast to the other primary tauopathies discussed thus far, Argyrophilic Grain Disease (AGD) lacks a well-defined clinical profile, largely due to its substantial overlap with other neuropathologies and the considerable heterogeneity inherent to the disease itself [86]. Similar to PSP or CBD, the overrepresentation of the *MAPT* H1 haplotype is observed in AGD [87]. Also, rare *MAPT* mutations relevant for AGD (e.g. S305I and S305S), as well as DNA copy number variations at 17p13.2 have been found [88–90]. AGD is a sporadic age-related tauopathy, it becomes more prevalent as individuals age, affecting approximately 40% of people aged between 90 and 100 years. Additionally AGD occurs in 25% of AD cases, contributing to the overall pathology [91–93]. The question arises whether AGD should be categorized as a neurological pathology or as a by-product of brain ageing, especially considering the prevalence of AGD hallmarks in the brains of healthy adults above 60 years old [86]. Although AGD falls within the 4R tauopathies, its hallmarks significantly diverge from those of PSP or CBD. AGD is distinguished by the presence of argyrophilic granules, from which it derives its name, as well as comma-shaped dendritic protrusions predominantly consisting of phosphorylated 4R Tau, which are crucially involved in the main neuropathologic features, the so-called “coiled bodies”, oligodendroglial TAU inclusions that go along with “ballooned” neurons [87, 94]. Primarily found in the hippocampus, amygdala, and adjacent temporal cortex, AGD less frequently affects regions such as the basal ganglia, brainstem nuclei, or cerebellum, which are more commonly involved in PSP or CBD [86]. AGD may exhibit a strong correlation with individuals experiencing late-onset prominent psychiatric symptoms. It is suspected to be a significant and isolated risk factor for psychiatric hospitalization and even completion of suicide [86].

For a comprehensive exploration of additional primary 4R tauopathies and their specific brain-related pathologies, refer to works by Chung et al. [20], Götz et al. [95], or Irwin [26].

### Other/rare primary tauopathies

In addition to the main primary tauopathies discussed above, there are other distinct tauopathies with underexplored genetic backgrounds. One such example is globular glial tauopathy (GGT), found within the TAU-positive FTLD-spectrum, representing less than 10% of FTLD-TAU cases [96]. For this assumed non-familial 4R tauopathy, only few cases have been linked to *MAPT* mutations (p.K317N, p.P301L) [97, 98]. The ageing-related TAU astrogliopathy (ARTAG) is rarely an isolated finding, but rather a co-pathology in ageing, with pathologic accumulation of abnormally phosphorylated 4R-TAU in astrocytes [24]. *MAPT* haplotypes (as well as *APOE* genotypes) do not have a significant effect on the presence of ARTAG, and other genetic causes for the development of the disease have not yet been sufficiently examined [99]. In the mixed 3R + 4R primary age-related tauopathy (PART), AD-type NFTs occur progressively in the absence of Amyloid co-pathology [100]. The cognitive impairment in PART seems to be mainly correlated to comorbid pathologies like cerebrovascular disease, but also typical *MAPT*-related FTD mutations like p.R406W and p.V337M are reported in PART pathogenesis [101, 102]. The *MAPT* H1 haplotype is again considered a strong risk factor for PART [103]. Interestingly, PART demonstrates that TAU dysfunction alone is sufficient to cause neurodegeneration, importantly contributing to the emergence of the term ‘primary tauopathies’ [104]. An overview of the primary tauopathies discussed here with regard to their genetic risk factors is given in Table 1.

**Table 1:** Primary tauopathies with brief clinical description, genetic factors, and major pathology

Disease entity	Clinic description/pathological overview	Genetic etiology	Primary pathology	References/overview
Ageing-related TAU astrogliopathy (ARTAG)	Majorly found in individuals above the age of 65. Common co-pathology, found in > 65% of primary tauopathy cases	*MAPT, AQP4*	Astrocytic lesions/Thornshaped astrocytes	[24] [105]
Argyrophilic grain disease (AGD)	Most common neurodegenerative disease after AD. No common clinical presentation or phenotype associated. Increased risk in older ages	*MAPT*	Argyrophilic grains especially within dendrites and spines	[106]
Corticobasal degeneration (CBD)	Average disease onset is 64 years, worsening in motor function and behavioral changes in patients	*MAPT*	4R TAU accumulation and pretangles found in neurons and glial cells	[107] [64] [60]
Globular glial tauopathy (GGT)	Average disease onset is 67 years. GGT manifests in three distinct subtypes, each characterized by a unique symptomatic profile. Symptoms include behavioral changes, cognitive impairment, and parkinsonism	*MAPT*	White matter degeneration featuring globular astrocytic inclusions prevails across all subtypes	[96] [97]
*MAPT*-related FTD/frontotemporal dementia with Parkinsonism-17 (FTDP-17)	Heterogeneous clinical presentation, but exhibits typical dementia symptoms like progressive cognitive decline, changes in behavior, memory loss	*MAPT*	Accumulation of filamentous and hyperphosphorylated TAU usually in both neurons and glial cells	[108] [109]
Progressive supranuclear palsy (PSP)	Motor impairment, many similarities with Parkinson’s disease (no tremor), specific PSP diagnosis challenging	*MAPT, LRRK2, DCTN1*	Astrocytic plaques especially in the cerebral cortex NFTs/tufted astrocytes and coiled bodies	[52] [110] [55]
Primary age-related tauopathy (PART)	Belongs to Alzheimer-type neurofibrillary degeneration, lacking Aβ-plaques	*MAPT*	NFTs indistinguishable from the early stages of AD	[102] [100]
Pick's disease (PiD)	Clinical presentation highly dependent on cortical atrophy location either resulting in Klüver-Bucy syndrome or frontal lobe syndrome. Many common symptoms with other dementia types as memory deficits or behavioral changes	*MAPT, PSEN1*	Pick bodies	[74] [82]

## Secondary tauopathies

The distinction between primary and secondary tauopathy is determined by whether the abnormal changes in TAU protein are the predominant pathology and likely the driver of disease or a co-pathology and possibly secondary to a TAU-independent disease cause. If pathological TAU formation develops in response to other pathogenic events and is not considered the driver of the disease, the term secondary tauopathy is used [12, 18]. In many tauopathies considered secondary tauopathies, TAU is the most important co-pathology and may be critical to the developing neurodegeneration, e.g. for AD [111], which makes a clear distinction in some cases disputable. The genetic factors leading to TAU pathology as well as the pathomechanistic connections between the primary pathology and TAU in secondary tauopathies are often not fully understood. This section provides an overview of the most relevant secondary tauopathies and the state of the art on the relationship between genetic predispositions and TAU pathology in the respective diseases.

### Alzheimer's disease (AD)

The classification of primary and secondary tauopathies can be challenging, as it is the case for Alzheimer’s disease. There are two main hypotheses behind the pathophysiology of the disease: the Amyloid hypothesis and the TAU hypothesis. The former hypothesis posits that the accumulation of Aβ oligomers is the main driver of the disease [112]. Mutations in the Amyloid precursor protein (APP), the precursor molecule whose proteolysis generates Aβ [113], or in Presenelin-1 or Presenelin-2, which comprise the catalytic domain of APP-protease γ-secretase [114], lead to increased or aberrant production and accumulation of Aβ-42. This is assumed to trigger a cascade leading to senile plaque formation, synaptic injury, oxidative stress, and altered cellular/enzymatic activities [115]. The hypothesis suggests that imbalance between Aβ production and clearance triggers TAU hyperphosphorylation and accumulation into NFTs [116]. According to the Aβ hypothesis, AD should be classified as a secondary tauopathy, since NFT formation is downstream of Amyloid pathology.

The TAU hypothesis on the other hand suggests a central role of TAU protein in driving the pathophysiology of AD. This hypothesis postulates that the (clinical) disease begins when TAU becomes hyperphosphorylated, leading to TAU dysfunction, e.g. due to TAU disassociation from and subsequent destabilization of the microtubules [117]. Hyperphosphorylated TAU missorts to somatodendritic compartments of neurons and aggregates into NFTs, culminating in disrupted axonal transport, synaptic loss, and neuronal death [9, 118]. In this hypothesis, TAU phosphorylation could also drive Aβ generation within afflicted neurons [119]. This would reclassify AD as a primary tauopathy where TAU is the main driver, and not just a mere bystander, of the disease. This hypothesis has recently been challenged by Aβ-based therapies, which showed a significant deceleration of disease progression also in sporadic AD cases [120], but it cannot be excluded that Aβ is only a modulator and not the primary disease driver.

However, the presence of both Amyloid plaques and TAU tangles is the defining feature of AD, giving rise to more holistic theories such as the neuroinflammation theory. Here, the persistent activation of the brain's microglia has been shown to exacerbate both Amyloid and TAU-related pathology, potentially playing a central role in the development of the disease [121]. Nonetheless, differential diagnosis of AD from other primary tauopathies, and primary tauopathies from each other can be challenging, as e.g. misdiagnosis of FTD as AD and vice versa is not uncommon. In principle, primary tauopathies can be distinguished based on the TAU isoforms present in the tangles, but these differences can only be seen in brain aggregates, making ante mortem distinction difficult [122]. A recent study reported several specific post-translational modifications (PTMs) on soluble TAU that can serve as signatures to distinguish between tauopathies. These include phosphorylation on Serine 184 coupled with phosphorylation on Serine 185 in the case of 3R-tauopathies, and ubiquitination on Lysine 343 in the case of 4R-tauopathies, but also more specific acetylation on Lysine 311 specific for Pick’s disease, ubiquitination on Lysine 369 specific for corticobasal degeneration, and ubiquitination on Lysine 31, 317, and 267 coupled with phosphorylation on Serine 262 specific for AD [123]. Unveiling these soluble TAU PTMs will help immensely in establishing fluid biomarkers and evaluating drug targets for tauopathies.

Although not more than 5–10% of AD cases are definitely caused by a single genetic mutation and *MAPT* mutations are generally not linked to familial forms of AD, more than 70 other genetic regions associated with AD have been identified [124] (extensively reviewed by Andrews et al. [125]). Several AD risk genes can influence TAU accumulation and phosphorylation. Apolipoprotein E4 (APOE4) raises TAU levels in the human brain and induces phosphorylation of TAU in human neurons, the *APOE* ε4 allele is considered the strongest risk factor for the development of AD and a lower age of onset [126]. *APP* also affects the accumulation of TAU, with the expression of APP increasing the number of phosphorylated TAU aggregates [127]. Other well-researched risk factors are mutations in both presenilin genes (*PSEN1* and *PSEN2*), which increase TAU phosphorylation and aggregation [128]. New machine learning-based approaches are of particular importance for the detection of genetic disease associations, such as the recent discovery of two new AD associated loci *SH3BP4* and *SASH1*, which also show significant epistatic interactions with *APOE*. [129]. In contrast, however, there are also protective genetic factors that reduce the risk of and represent a resilience to AD (reviewed in detail in Seto et al. [130]). These findings enable new, promising approaches for AD therapy options, as a recent study showed that Tripartite motif-containing protein 11 (TRIM11) is downregulated in AD and that AAV-based viral delivery of *TRIM11* confers strong protection against TAU pathology in several mouse models [46]. However, it is still mostly unknown how genetic risk factors can influence the progression and transmission of TAU pathology. A less known genetic risk factor for AD, *FRMD4A*, has been shown to affect TAU secretion, and is thought to modulate TAU release as well as cell-to-cell transmission in AD via the FRMD4A-cytohesin-Arf6 presynaptic vesicle pathway [131].

While for rare (genetic) tauopathies epigenetic studies are largely missing (but see chapter PSP for *MAPT* promoter hypomethylation), for the mainly sporadic AD and PD, epigenetic factors are certainly involved, and may play a disease-modifying role also in other (genetic) tauopathies, e.g. modifying the age of onset or severity of symptoms, and also be consequence of gene mutation (as e.g. discussed here for myotonic dystrophy, which changes splicing in a wide-spread fashion, see below). While this is outside the scope of this review, epigenetics in AD and other tauopathies are reviewed in Zimmer-Bensch and Zempel [2]. In AD, global DNA hypomethylation in postmortem tissue, also confirmed by monozygotic twin studies, but also differential methylation of specific genes like *ANK1, MCF2L, STK32C, LRRC8B, MAP2*, and *S100B*, all associated with neuronal function, as well as methylation changes at key AD risk genes such as *APP* and *ADAM17* have been reported, but also other epigenetic changes and also changes in factors upstream of TAU pathology (e.g. CDK5, GSK-3 beta) [2].

While naturally therapeutic strategies for AD are primarily focused on Amyloid-beta (with some clinical success) and TAU, the range of biological, pharmacological and psychological treatments spans conformation specific antibodies over treating underlying diabetes and inflammation as well as exercise and social activities, with literally billions invested in clinical studies and industry- and academia-based research [44]. Apart from highly disease-specific interventions, there is reasonable hope that treatment of underlying problems like e.g. obesity, diabetes or heart disease which is beneficial to prevent/delay the onset of Alzheimer’s or dementia in general, may also delay or slightly alleviate genetic forms of tauopathies.

In sum, elucidating the complete cascade of AD pathology, and distinguishing it from other primary tauopathies is still challenging. While TAU-based therapeutic strategies are certainly valid (see e.g. Al Kabbani et al. [132]), more insights in the future will help to develop better biomarkers to differentially diagnose and monitor these diseases, and will unveil more specific and genetically validated therapeutic targets.

### Lewy body dementia (LBD)

Lewy body dementia (LBD) comprises both dementia with Lewy bodies (DLB) and Parkinson’s disease (PD). PD is histopathologically characterized by the progressive loss of dopaminergic neurons in the substantia nigra. The main pathological hallmark of PD is the formation of Lewy bodies (LBs) consisting of crowded organelles, lipid membranes, and aggregated α-synuclein in the remaining neurons [133, 134]. TAU pathology (e.g. ectopic expression, aggregation, phosphorylation) is frequently observed in the synucleinopathies PD and DLB, but a clear cause-effect between misfolded protein aggregates and neurodegeneration has not been demonstrated [67]. After the discovery of co-localization of α-synuclein and TAU [135], many studies explored the mechanisms of how TAU contributes to the pathophysiology of PD. Around 80% of PD patients develop Parkinson’s disease dementia (PDD) during their disease course [136]. AD-type pathology, i.e. NFTs, and Aβ plaques, are positively correlated with cognitive impairment in PDD [137, 138]. Hence, TAU is potentially a protagonist in the development of dementia following PD.

Mutations in the leucine-rich repeat kinase 2 (*LRRK2*) are the most common genetic cause of Parkinson’s disease. LBs, the major feature of idiopathic PD (iPD), are not found in all *LRRK2* PD cases, suggesting that in *LRRK2*-associated PD there is another driver of the disease. Strikingly, TAU pathology (AD-type TAU tangles and/or abnormal TAU phosphorylation) is found in the majority of *LRRK2* PD cases [139]. This suggests a relationship between *LRRK2* mutations and the development of TAU pathology. Mutations in *LRRK2* are also associated with pathologically confirmed primary tauopathies, such as PSP or CBD [140]. In addition, certain *MAPT* mutations are reported to be rare causes of primary tauopathies considered as atypical parkinsonism syndromes (e.g. PSP and CBD) [67]. The *MAPT* H1/H1 haplotype, which is associated with increased risk of some primary tauopathies (especially PSP), has been associated with PD as well [138, 141]. Even though homozygosity for the *MAPT* haplotype H1 is associated with increased risk for PD, a recent post-mortem study could not identify a link between the H1/H1 associated overexpression of *MAPT* and PD status [142]. Many other genes may be risk factors for PD because the proteins they encode interact with TAU, and mutations in these genes have been described to affect TAU pathology in PD cases. The most common of these genes include, among many others with lower frequency, *PINK1* [143], *SNCA* [144], *GBA* [145]*,* and *PARK7* [146] (see also Vacchi et al. [147] for a comprehensive overview).

Dementia with Lewy Bodies (DLB) is clinically similar to PD and characterized by the accumulation of aggregated α-synuclein in Lewy bodies and associated with loss of nigrostriatal dopaminergic neurons. Additional pathologies include hyperphosphorylated TAU and AD-like neurofibrillary tangles [148]. There is no sharp distinction between PDD and DLB in terms of neuropathology, the most significant difference between phenotypes PDD and DLB is the degree of AD-like pathology in terms of plaque deposition, which is higher in DLB than PDD [149, 150]. A substantial proportion of DLB patients have abnormal values for the biomarkers CSF Aβ42, total TAU, and phosphorylated TAU, a profile which is more common in DLB compared to PD/PDD, and associated with more severe cognitive impairment in DLB [151]. Especially the Casp2-generated TAU fragment Δtau314 (a soluble form of TAU) is connected to dementia in DLB. Levels of Δtau314 are around twice as high in DLB relative to PD, which could hint towards a TAU-based or TAU-related mechanism for synaptic dysfunction underlying dementia in DLB [152]. Many genetic variants are associated with DLB (reviewed in Outeiro et al. [148] and Tolea et al. [153]). The strongest and most replicated ones are the *APOE* ε4 allele (also the strongest risk factor for AD) and *GBA*, both likely involved in the mechanism of LB pathology formation and/or spread [148, 154, 155]. Other reported risk genes of DLB are *SNCA, SCARB2,*

*PSEN1, PSEN2*, and *MAPT* [156, 157]. Although overrepresentation of the *MAPT* p.A152T variant, H1/H1 haplotype, and H1g subhaplotype are considered potential risk factors for DLB, overall current evidence suggests that *MAPT* variations may only have a minor role in DLB [67]. Strikingly, a recently discovered new risk loci, *BIN1*, was significantly associated with increased NFT pathology in DLB [158]. Loss of *BIN1* is known to impair synaptic transmission and promotes the propagation of TAU pathology [159, 160].

In genetic forms of DLB/PD, epigenetic factors may play a role in disease modifications, but are likely not the driving force of disease progression. In sporadic DLB/PD, however, epigenetic changes may be disease drivers, and give interesting hints towards disease mechanisms. While *MAPT* methylation can be both increased and decreased, the genes/loci responsible for the expression of α-synuclein and PGC1-α (key factor in mitochondrial biogenesis) are hypo- and hypermethylated, respectively (for review see Zhang et al. [161]), indicating a crucial role for α-synuclein but also mitochondria in DLB/PD. As mitochondrial function must be critically involved in the genetic forms of PD associated with mitochondrial genes, and the (epi-) genetic and histopathological evidence pointing towards α-synuclein, bolstering of mitochondria and targeting alpha-synuclein are natural targets. While mitochondrial bolstering (e.g. via MitoQ and Q10) were unsuccessful in clinical studies (see Borsche et al. [162] for review), α-synuclein-based treatments show considerable effects, and trials are ongoing [163]. Naturally, gene therapy approaches are considered for genetic forms of PD (with clinical trials ongoing for *GBA1* or *LRRK2*), but conventional treatments (e.g. levodopa) and deep brain stimulation are effective and the most widely used treatment also for PD [164].

Collectively, both DLB and PD are defined by widespread α-synuclein pathology, but AD-like TAU pathology might as well contribute to the development of dementia.

### Niemann-Pick disease type C (NPC)

The rare lysosomal storage disorder Niemann-Pick disease type C (NPC) is caused by mutations in the *NPC1* (95%) or *NPC2* (5%) gene. Patients present with progressive neurodegeneration, resulting clinically in vertical supranuclear palsy, dysarthria, and dysphagia [165]. This is very different from Niemann-Pick disease type A or B, which usually are more associated with liver and lung involvement and have not been associated with TAU pathology to date [166]. Histopathologically, NPC is primarily characterized by extensive accumulation of cholesterol in several tissues, including the brain [167]. Brains of adult NPC patients exhibit AD-like TAU protein hyperphosphorylation and neurofibrillary tangles [165, 168]. Despite parallels concerning cognitive impairment and cellular pathology of NPC and AD, genetic variants and polymorphisms in *NPC1* and *NPC2* are not directly associated with elevated AD risk in the Chinese population [169], and *NPC1/2* variants did not confer susceptibility for several Tauopathies (PD, FTLD-TAU, PSP) [170, 171]. Pathomechanistically, functional TAU protein is critical to the induction of autophagy in NPC1 deficiency. The hyperphosphorylation of TAU leads to a progressive loss of its normal protein function and impairs both autophagic flux and induction in NPC1-deficient models, but not in healthy cells [172]. This suggests a bidirectional mode of action, where disturbances in cellular cholesterol metabolism may promote TAU pathology, but abnormal TAU also alters neuronal cholesterol homeostasis, leading to a vicious cycle eventually resulting in cholesterol accumulation and NFT formation [173, 174]. Thus, pathogenic *NPC1* and *NPC2* mutations are the causative agents for NPC but do not represent a general risk factor for other tauopathies. In fact, both *MAPT* knockout and *MAPT* haploinsufficiency were associated with decreased survival in mice mimicking NPC [175, 176]. This indicates that TAU may even have a protective role in NPC, which contrasts TAUs usual role of a disease-driving factor in other tauopathies.

### Down syndrome (DS)

Down syndrome/trisomy 21 (DS) is a multisystemic disorder caused by an extra copy of a critical region on or the entire chromosome 21. DS is associated with developmental delay, intellectual disability, and characteristic morphological and syndromic features, but is also considered a form of genetically determined AD [177]. First clinical symptoms in terms of cognitive decline often appear in the fourth decade of life with a > 90% lifetime risk to develop dementia [178, 179]. DS patients have a high prevalence of AD-like dementia with Aβ and TAU pathology. Regions where TAU often accumulates in tauopathies (e.g. medial and basal temporal lobe) show high levels of cortical atrophy [179]. The distribution of Aβ plaques and NFTs is observed to be very similar in DS and AD, but the density is greater in DS [180, 181]. Recently, neuropathological differences between DS and sporadic AD have been reported, including the morphology of Aβ plaques and the distribution of NFTs [182, 183]. Genetically, the cause of early onset dementia in DS is a 1.5-fold increase in Aβ production and early Aβ plaque disposition due to the localization of the *APP* gene on the triplicated chromosome 21 [178, 184]. The increased expression of not only *APP*, but also others of the more than 310 genes on chromosome 21 may contribute to and likely modulate AD-like dementia pathology in the diverse DS phenotypes [185, 186]. A functional link between DS and AD is the overexpression of DYRK1A protein due to the extra copy of the *DYRK1A* gene on chromosome 21 (which also maps to the critical region of AD). DYRK1A is a kinase that potentially phosphorylates or interacts with several proteins, e.g. transcription factors, and also mediates hyperphosphorylation of TAU [187]. Despite classification of DS as a secondary tauopathy due to predominant Aβ pathology, TAU is a significant predictor of cognitive and functional decline in DS-related dementia, independent of Aβ deposition [188]. Neurofibrillary TAU already occurs in individuals with DS as early as Braak stages I-II (with very low Amyloid burden), not only in stages III-VI with higher Amyloid burden [189]. Strikingly, overproduction of Aβ leads to an upregulation of DYRK1A levels and subsequent TAU phosphorylation, making DYRK1A a potential key molecule in the vicious cycle of Aβ production and TAU hyperphosphorylation in DS [190, 191]. Increased dosage of DYRK1A regulates alternative splicing of the *MAPT* gene in DS by phosphorylating the alternative splicing factor (ASF), preventing it from facilitating exon 10 inclusion. This correlates to a relative increase in 3R-TAU levels, leading to imbalance of 3R - and 4R-TAU in DS brain, facilitating formation of neurofibrillary degeneration and making DS a rare example of a 3R-predominant tauopathy [192]. The inhibition of DYRK1A (e.g. with CX-4945) potentially suppresses the aberrant phosphorylation of TAU, therefore this may be a strategy for disease modification in DS [193, 194]. DYRK1A inhibitors are also investigated as potential candidates to counteract AD, targeted DYRK1A inhibition rescued the AD-typical phenotype in several models (Drosophila, APP/PS1 mice) [195, 196]. A recent study associates TAU pathology in DS with the retromer complex system, as pathogenic TAU negatively correlates with retromer proteins and cathepsin-D activity, which might contribute TAU accumulation. This suggests the retromer complex as another potential regulator of pathogenic TAU in DS [197]. In sum, dementia in DS is mainly caused by increased expression of *APP* and other genes present in the DS-critical region of chromosome 21 (e.g. *DYRK1A*). TAU is indirectly impacted via APP/Aβ and directly via DYRK1A (due to aberrant splicing and phosphorylation) and possibly others, making it a likely disease driver. Hence, TAU-based therapeutic strategies may be beneficial also in DS.

### Myotonic dystrophy (DM)

The neurodegenerative disease Myotonic dystrophy is found in two manifestations, Myotonic dystrophy Type 1 and 2 (DM1 and DM2). DM1 is a multisystemic neuromuscular disease with cognitive dysfunction [198]. In DM1, CTG microsatellite repeat expansion in the 3’ untranslated region of dystrophia myotonica protein kinase (*DMPK*) gene induces the expression of toxic RNA aggregates (nuclear foci), which is thought to cause aberrant splicing of various genes, leading to multisystemic symptoms in various tissues, including heterogeneous brain involvement [199, 200]. NFTs in DM1 show a preferential accumulation of the 0N3R TAU isoform due to a modified splicing pattern of *MAPT*, mainly characterized by the reduced inclusion of exons 2 and 3 [201–203]. Altered splicing of *MAPT* exon 10, likely a consequence of a gain of embryonic lethal abnormal vision-like RNA-binding protein-3 (ETR3/CELF2) function, occurs [202, 204]. The dysregulation of alternative splicing possibly leads to pathologic TAU protein accumulation/NFTs and might be a critical pathological characteristic in the brain of DM1 patients [200, 205]. The TAU pathology in DM1 patients is highly variable and different to the one in AD [206]. The cognitive phenotype in DM1 is caused by nuclear foci and resulting aberrant splicing of not only *MAPT*, but numerous other pre-messenger RNAs (reviewed by López-Martínez et al. [207]). Noteworthy, abnormal expression of BIN1 was reported in DM1, which may be associated with NFT pathology (similar to DLB) [205, 208]. The distribution of neuropathological changes does not correlate with the length of CTG repeats in the *DMPK* gene, which favors a more multifactorial pathomechanism in DM1 [209]. This is also indicated by the discovery of a widespread Lewy body pathology in DM1 in addition to TAU pathology, which does not follow the Braak classification [210]. The extent to which TAU contributes to neuronal pathology in DM1 is uncertain because TAU missplicing also occurs in non-TAU proteinopathies, and neurodegeneration in DM1 cannot be clearly linked to aberrant TAU [203]. Overall, due to the frequent observation of NFTs in DM1 brains and a clear genetic cause (*DMPK* mutation), DM1 can be classified a secondary tauopathy, despite the apparent missplicing of *MAPT*.

In general, DM2 is more a muscle disease with less multisystem and central nervous system involvement compared to DM1. However, the cognitive impairment is comparable to DM1 but much less severe [211]. Genetically, it is caused by an unstable CCTG repeat expansion in the nucleic acid-binding protein (*CNBP*) gene [212]. Histopathology in DM2 has consistently shown TAU pathology in the brain similar to that in DM1 patients [213], justifying a classification as a genetic secondary tauopathy, but detailed pathomechanistic studies are still lacking.

### Huntington’s disease (HD)

The autosomal dominant neurodegenerative disorder Huntington’s disease (HD) is characterized by severe motor, cognitive and psychiatric deficits in its final disease stage. Causative is an expanded CAG repeat in exon 1 of the *HTT* gene, leading to the expression of abnormally modified huntingtin (HTT) protein, which aggregates in nearly all cells of the body of HD patients and is the primary pathogenic event in HD [214, 215]. The mutant HTT protein interacts with β-tubulin and binds to microtubules. Since HD patients show brain-wide aggregated TAU inclusions, HD is classified as a secondary tauopathy [216]. The haplotype of *MAPT* influences the clinical picture in HD, patients with the H2 *MAPT* haplotype present a more rapid cognitive decline compared to the H1 carriers, indicating a disease driving role for TAU in HD [217, 218]. Hence, therapeutic approaches for the treatment of HD besides targeting HTT comprise targeting of TAU similar to AD, including modulation of *MAPT* gene expression, inhibition of TAU aggregation, targeting hyperphosphorylated TAU, and TAU immunotherapies [219]. Differential regulation of *MAPT* exons 2, 3 and 10 was observed in various brain regions in HD [220]. An imbalance of TAU isoforms (especially the 4R/3R ratio) in favor of the 4R isoform due to altered *MAPT* splicing (increased exon 10 inclusion) is reported in HD similar to a subset of primary tauopathies. Yet, little is known about the implications of TAU in the pathophysiology of HD and whether pathogenic *HTT* mutations lead to dysregulation of TAU, as HTT and TAU do not directly interact with each other [221]. Potential indirect connections between mutant HTT and dysregulated TAU include various TAU kinases, the serine/arginine-rich splicing factor 6 (SRSF6), and the DNA/RNA binding fused in sarcoma protein (FUS) [222]. Evidence for an important role of TAU in the neurodegenerative pathology of HD is the recent finding that passive immunization against phosphorylated TAU decreases Amyloid fibrils and huntingtin oligomers in mice [223]. Although TAU may be a driver of pathogenic changes in HD as it is hyperphosphorylated already in early disease stages, modulation of TAU expression in HD mice does not influence the progression of the HD phenotypes [224, 225]. For review on the implications of TAU dysregulation in HD see Mees et al. [222] and Salem and Cicchetti [217]. In sum, while there is evidence for a role of TAU as a contributor or even disease driver, it may not be an ideal therapeutic target for HD.

### Other/rare secondary tauopathies

There are other hereditary diseases that can be classified as genetic tauopathies and are likely secondary as the genetic cause of these diseases has not been shown to be directly related to TAU physiology or pathology. Most of these disorders are rare and sparsely studied, providing no evidence of a link of the underlying disease-causing gene-mutations and the observed TAU pathology. However, for some cases, there are compelling indications for a link between disease cause and TAU abnormalities. Among these are familial British (FBD) and familial Danish dementia (FDD), neurodegenerative conditions considered hereditary types of cerebral Amyloid angiopathy (CAA). This disease spectrum is mostly caused by *APP* mutations and predominantly characterized by Aβ pathology [226, 227]. In some cases of CAA, there is significant TAU pathology, such as TAU aggregation in brains of FBD/FDD affected individuals [228]. Causative for FBD/FDD are mutations on the human integral membrane protein 2B (*ITM2b*) gene, encoding BRI2, leading to production of 34 amino acids non-Aβ Amyloids that are neurotoxic [227]. Strikingly, mutant BRI2 contributes to changes in TAU metabolism and synaptic dysfunction [229], the BRICHOS domain of the protein inhibits Aβ aggregation and regulates the initiation of the Amyloid cascade, including truncation of TAU [230]. Another example is the neurodevelopmental Christianson Syndrome, which is caused by loss-of-function mutations in the X-linked *SLC9A6* gene, encoding for the endosomal Na + /H + exchanger 6 (NHE6). NHE6 knockout human neurons show elevated phosphorylated and sarkosyl-insoluble TAU [231]. The TAU and Aβ aggregation in Christianson Syndrome may be preceded by early lysosome defects, which suggests linkages between endolysosomal dysfunction and AD-like neurodegeneration and is in line with basic studies on this pathway [232, 233]. Some genes are suspected or confirmed to contribute to TAU pathology, but are definitely not the sole cause of the known disease. These include for example the tuberous sclerosis complex-1 (*TSC1*) gene, which in recent years has been associated with TAU pathology in frontotemporal dementia and Alzheimer's disease, where loss-of-function TSC1 inclusions lead to an increase of TAU burden [234–236]. In addition, diseases not previously described as genetic tauopathies can be (re-)classified as such once patients with TAU pathology are reported. Most recently, this is the case for ocular pharyngeal muscular dystrophy, where mutations of the *PABPN1* gene cause loss-of-function of the poly(A) RNA binding protein, which has been demonstrated to cause accumulation of pathological TAU [237]. Pathogenic mutations in the prion protein gene (*PRNP*) cause genetic Creutzfeldt-Jakob disease (gCJD), a disease that shares several neuropathological features with other neurogenetic disorders like AD and PD, including TAU pathology [238]. TAU was identified as a regulator of *PRNP-*transcription and actively upregulates the expression of the gene product cellular prion protein (PrPc) [239]. Additionally, *PRNP* mutations are known to be a rare cause in the FTD spectrum, in line also with overlapping clinical features of prion disease and FTD [240]. Similarly, the rare Gerstmann-Sträussler-Scheinker disease (GSS), a neurodegenerative disease with severe dementia and late-stage neurodegeneration, is triggered by pathogenic variants of the *PRNP* gene [241]. TAU deposits can be detected in different brain regions of GSS patients [242].

The transactivation response DNA binding protein 43 kDa (TDP-43) is a RNA-binding protein of which ubiquitylated aggregates are observed in the neurons of ALS and FTLD patients [243]. There are some hints for pathomechanistic links between TDP-43 proteinopathies (comprising e.g. familial FTLD with TDP-43 pathology (FTLD-TDP), caused by mutations in *GRN* or *C9orf72* hexanucleotide repeat expansion) and tauopathies, as e.g. the TAU tubulin kinases TTBK1/2 promote accumulation of pathological TDP-43 [244]. Recently, concurrent TAU pathology in FTLD-TDP with TDP-43 pathology was observed [245]. There is also an association of TDP-43 pathology with increased TAU burden and worsened p-TAU aggregation [246]. However, despite TAU pathology in subtypes of FTLD-TDP, there is no evidence yet for genetic factors impacting TAU accumulation in this spectrum of diseases. In patients affected by frontotemporal dementia and/or amyotrophic lateral sclerosis-6 (FTDALS6), caused by mutations in the Valosin-containing protein (VCP), there is also TAU pathology and deposition of pathologic TDP-43. However, in this case the disease-causing *VCP* mutation p.Asp395Gly is linked to the aggregation propensity of TAU, as it impairs the TAU disaggregase activity of VCP, making it a potential new therapeutic target for AD and other tauopathies [247].

Amyotrophic lateral sclerosis-Parkinsonism/Dementia complex-1 is a rare neurodegenerative disease with NFT pathology primarily found among Chamorros, the indigenous people of Guam. Not only SNPs in *MAPT*, but also the T1482I variant in the *TRPM7* gene (encoding for the ion channel TRPM7) may confer susceptibility to disease development [248]. The resulting mutant TRPM7 shows increased sensitivity to intracellular Mg^2+^- a remarkable gene-environment relationship, as the incidence of the disease has been associated to long-term exposure to a Mg^2+^ -deficient environment (see also chapter below) [249]. Interestingly, recent findings provide a mechanistic link between loss of TRPM7 and promoted Aβ degradation in AD, but no direct association with TAU pathology was found so far [250]. A very rare form of parkinsonism (X-linked parkinsonism with spasticity, XPDS) shows pathological 4R TAU deposits and is caused by altered splicing of the ATPase H(+) transporting lysosomal accessory protein 2 (*ATP6AP2*) gene, which causes lysosomal dysfunction in XPDS [251, 252]. A group of inherited diseases called neurodegeneration with brain iron accumulation (NBIA), characterized by cognitive and behavioral changes and parkinsonism, comprises mitochondrial membrane protein-associated neurodegeneration (MPAN or NBIA4). This condition, caused by pathogenic sequence variants in *C19orf12*, was reported as a secondary tauopathy, as TAU pathology was reported by several studies [253–255]. Recently, TAU pathology was observed in some cases of spinocerebellar ataxia type 8 (SCA8), a condition caused by abnormal CTA/CTG repeat expansions in *ATXN8OS* which have also been reported in PSP [256]. However, a precise pathomechanistic connection to TAU has not yet been established in these cases.

Generally, the less prevalent a mutation and the less it is understood to be the primary cause of a neurodegenerative disease, the more difficult is it to attribute TAU pathology to this mutation in a direct pathomechanistic way. If - as in the recently discovered exemplary cases of genetic disease due to *TSC1* or *PABPN1* mutation - there is a convincing clinical and experimental rationale demonstrating that these mutations may have direct effects on TAU homeostasis, we can assume a pathomechanistic connection, which could also be therapeutically leveraged. In case of *TSC1*, this has already been done, as rapamycin (a drug inhibiting mTOR, which is in turn activated by TSC1/2) was used in numerous studies for AD/tauopathies, but with ambiguous results [257].

### Sporadic secondary tauopathies

There are few non-genetic and sporadic diseases, e.g. triggered by infections that are also associated with TAU pathology. In many cases, however, the TAU pathology is likely a consequence of the preceding conditions, as in the case of subacute sclerosing panencephalitis (SSPE). Here, tauopathy is a consequence of an infection with a mutated measles virus resulting in diffuse brain inflammation [258]. There are also well characterized genetic predispositions for initially completely sporadic appearing clinical entities. Some genetic prion diseases (gPrDs) may be genetically associated with other neurodegenerative diseases, as abnormalities of particular genes (e.g. *LPA, LRRK2, TET1, FGF20, ACO1, *and *POSTN*) have been linked to both gPrDs and AD or PD [238]. In a subgroup of tauopathies, including traumatic brain injury (TBI), mechanically evoked traumatic axon injury precedes TAU pathology and NFT formation [259]. The suffering of a head trauma/TBI induces TAU hyperphosphorylation and aggregation. Moderate to severe TBI can trigger the initial formation of pathological TAU, starting the deadly cascade of TAU-related neurodegeneration [260]. Repetitive head trauma may trigger the long-term neurodegenerative process chronic traumatic encephalopathy (CTE), characterized by progressive neurodegeneration with hyperphosphorylated TAU [261]. Although in these cases the triggers of disease are defined and non-genetic, the prognosis and the brains ability to regenerate are genetically influenced. Recent GWAS studies have unveiled new potentially protective genes which, however, require validation and further analysis, especially since the direct correlation to the improvement of TAU pathology has not been proven yet (for review see Cortes and Pera [262]). Also experimental evidence shows that at least murine TAU may be protective in some settings of sporadic secondary tauopathy, as TAU KO mice fared worse in paradigms of TBI [263]. Outcome is also improved, if TAU is present and specific PTMs of TAU (e.g. acetylation of TAU) are pharmacologically repressed via inhibition of upstream acetylases [264]. First reported in 2014, the Anti-IgLON5 disease is a rare autoimmune disorder of the nervous system manifesting with cognitive impairment, gait instability, bulbar syndrome, and sleep and movement disorders [265, 266]. The disease is connected to autoantibodies against the neuronal cell adhesion protein IgLON5, which may be involved in the development and regulation of the central nervous system [265, 267]. Little is known about the pathogenic mechanisms resulting in neurodegeneration, but cellular investigations and experiments in mice suggest an antibody-mediated pathogenesis, with anti-IgLON5 antibodies as the main disease cause leading to irreversible cognitive and behavioral deficits [268]. Post-mortem examination of IgLON5 patient brains demonstrate neuronal loss with the presence of not only anti-IgLON5 antibodies but also deposits of hyperphosphorylated TAU (both 3R and 4R), predominantly in the hypothalamus, tegmentum of the brainstem, hippocampus, and cerebellum [269, 270]. TAU depositions in anti-IgLON5 disease patients visualized with a dynamic PET scan show increased [18F]PI-2620 TAU binding potentials in the pons, dorsal medulla, and cerebellum [271]. A recent study indicates that anti-IgLON5 antibodies precede the TAU pathology and suggests that TAU pathology occurs in later disease stages where it may also present like a PSP neuropathological phenotype with exclusively 4R neuronal and glial TAU pathology [272]. Genetically, there is a strong association between disease and the presence of human leukocyte antigen (HLA) alleles HLA-DRB1*10:01 and HLA-DQB1*05:01. This supports a primary autoimmune origin, but a significant association of *MAPT* H1/H1 homozygous haplotype was observed as well [273]. Hence, while IgLON5 is considered a sporadic disease and can be classified as a secondary tauopathy, TAU may play an important role as disease driver.

Environmental factors, regardless of genetic defects, can have a noticeable impact on post-translational modifications of TAU and therefore leads to environment-induced sporadic tauopathies. For example, zinc exposure leading to increased level of zinc ions in the brain is able to induce TAU oligomerization and ultimately the formation of paired helical filaments (PHFs) [274]. Another example of the implication of metals in tauopathies is the PSP-like tauopathy clusters observed in regions surrounding chemical plants, where the soil is heavily contaminated with heavy metals such as arsenic and chromate [275].

Rather peculiar examples of environmental exposure-induced tauopathies are geographically isolated PSP-like tauopathies, such as Guam parkinsonism-dementia complex (ALS-PDC) and Guadeloupean parkinsonism. The former is prevalent among the Chamorro population of the island of Guam [276], and is thought to emerge from exposure to the neurotoxin β-Methylamino-L-alanine enriched in the seeds of the cycad plant, a popular food on Guam [277]. While Guadeloupean parkinsonism shares a similar clinical picture with Guam ALS-PDC, the prevalence of the disease is thought to stem from the indigenous tradition of ingesting herbal tea and tropical fruits from the soursop and sugar apple plants, rich in neurotoxic benzyltetrahydroisoquinoline alkaloids [278]. While patients of both diseases exhibit NFT pathology and hyperphosphorylated TAU in several brain regions, there are no *MAPT* mutations associated with these diseases.

To conclude, in some sporadic neurodegenerative diseases, TAU pathology is secondary to another pathogenic event, but TAU may contribute to the disease progression, and may for some diseases constitute an attractive therapeutic target. A comprehensive list of genetic secondary tauopathies is provided (Table 2).

**Table 2:** Secondary tauopathies with brief clinical description, genetic factors and primary pathology

Disease entity	Clinic description/pathological overview	Genetic etiology/contributors in case of genetic disease	Primary pathology	References/overview
Parkinson's disease (PD)	α-synucleinopathy with motor symptoms, tremor, loss of postural reflexes, in PDD: progressive dementia	*LRRK2, PINK1, SNCA, GBA, PARK7, MAPT*	Lewy bodies (α-synuclein deposits)	[139]
Dementia with Lewy bodies (DLB)	α-synucleinopathy with impaired movement, cognitive decline, memory loss	*APOE, GBA, SNCA, SCARB2, PSEN1, PSEN2, MAPT, BIN1*	Lewy bodies (α-synuclein deposits)	[148] [153] [158]
Niemann Pick disease type C (NPC)	Lysosomal storage disease with hepatosplenomegaly, progressive dementia and premature death ranging from infancy to late adulthood	*NPC1, NPC2*	Cholesterol accumulations	[165] [170] [172]
Down syndrome (DS)	Developmental delay, intellectual disability, dementia over age of ~ 40	*APP, DYRK1A* (Chromosome 21 trisomy)	Amyloid-pathology	[178] [190] [192]
Myotonic dystrophy type 1 (DM1)	Myotonia, progressive muscular atrophy and pareses, includes cataracts, cardiac involvement, diabetes, and dementia	*DMPK*	Nuclear RNA foci	[205]
Huntington's disease (HD)	Severe motor, cognitive and psychiatric deficits	*HTT*	Huntingtin protein aggregates	[216] [221]
Cerebral amyloid angiopathy (CAA)	Cognitive decline, hemorrhagic stroke, small vessel vasculopathy	*APP, PRNP*	Amyloid-pathology	[226]
Hereditary cerebral amyloid angiopathy (familial british/danish demetia)	Familial forms of dementia (fam. British and fam. Danish dementia	*ITM2B*	Amyloid-pathology	[227]
Christianson syndrome	Postnatal microcephaly, developmental delay, aphasia, epilepsy and progressive cerebellar ataxia	*SLC9A6*	Endolysosomal dysfunction	[231] [232]
Ocular pharyngeal muscular dystrophy	Ptosis and dysphagia, proximal pareses, TAU neuropathology	*PABPN1*	PABPN1 loss of function	[237]
Familial Alzheimer disease	Age of onset usually between 40 and 70 years, fast progression	up to ~ 75 risk modifying genes, including* APOE, APP, PSEN1, PSEN2,*	Amyloid-pathology	[124] [125] [126] [128] [129]
Frontotemporal lobar degeneration with TDP43 inclusions (FTLD-TDP)	Neuronal loss in the superficial layers of the frontotemporal cortex, gliosis	*GRN*	TDP-43 inclusions	[244] [246]
Amyotrophic lateral sclerosis-Parkinsonism/Dementia complex-1	Neurodegenerative disease with high incidience on Guam, phenotype similar to PD associated with *PARK7* mutations	*TRPM7*	NFT pathology	[248] [249]
X-linked parkinsonism with spasticity (XPDS)	Classic parkinsonism, spasticity	*ATP6AP2*	Lysosomal dysfunction	[251] [252]
Frontotemporal dementia and/or amyotrophic lateral sclerosis-6 (FTDALS6)	Highly variable, e.g. progressive neurodegeneration, motor impairment	*VCP*	Ubiquitin-positive inclusions and TDP-43 inclusions	[247]
Neurodegeneration with brain iron accumulation 4 (NBIA4/MPAN)	Cognitive and behavioral changes, parkinsonism, slow progression	*C19orf12*	Brain iron accumulation	[253] [254] [255]
Spinocerebellar ataxia type 8 (SCA8)	Cerebellar ataxia, parkinsonism, cognitive impairment	*ATXN8OS*	Nuclear RNA foci	[256]
Creutzfeldt-Jakob disease (CJD)	AD- and PD-like neuropathology	*PRNP*	Accumulation of misfolded prion protein (PrP)	[238]
Gerstmann-Straussler disease (GSD)	Cerebellar ataxia, dysarthria, memory impairment, cognitive dysfunction	*PRNP*	Accumulation of misfolded prion protein (PrP)	[242]

## Diagnostic and therapeutic considerations

Misdiagnosis is not uncommon in particular in late-onset neurodegenerative diseases, which is arguably the biggest group of patients for tauopathies. Because of the well known clinical heterogeneity for some tauopathies, that can span e.g. from Amyotrophic Lateral Sclerosis to the very different and neurologically/phenotypically clearly separated FTD also in the same families for mutations/repeat expansions in the gene *C9orf72*, and e.g. *PSEN2*, mutations, we do not recommend single-gene diagnostics for the diseases discussed here. Rather, as is common standard of care in human genetics and neurology, and which is also justified by the many possible atypical manifestations, we believe that exome/genome-based virtual multi-gene panel or an unbiased approach is the most suitable way to genetically confirm a clinical diagnosis [279, 280]. Human geneticists and neurologists are aware that one gene can cause multiple disease phenotypes as outlined also in Tables 1 and 2, and that genotype–phenotype correlation can vary considerably also in the same family. Further, while tauopathy can now be detected in a fairly confident manner e.g. using PET-imaging and CSF-analysis and these clinical exams could be upstream of a genetic analysis, this is uncommon for diseases that manifest e.g. with a neuromuscular phenotype during childhood or early adult. If, however, tauopathy is suspected, the genes listed here can serve as an addition to phenotype-based multigene panel exome/genome analysis. Apart from TAU, in case of secondary tauopathies the causative gene is naturally the prime target for (gene) therapy considerations. Already now there are several clinical trials ongoing or recruiting for genetic tauopathies, which include (but are not limited to) also interventional studies e.g. *MAPT, SOD1, HTT, GRN, PRNP, TSC1*, but may not be available regionally. If a genetic cause has been identified, we strongly recommend to verify important clinical trials registries (like clinicaltrials.gov, euclinicaltrials.eu or clinicaltrialsregister.eu) for regionally available clinical studies.

## Conclusion

Epigenetic modifications, missense mutations, and rarely deletions in the *MAPT* gene are involved only in a fraction of tauopathies. If *MAPT*/TAU itself is the causative agent of a tauopathy and/or its primary pathological hallmark, we consider the disease a primary tauopathy. In the case of hereditary secondary tauopathies, *MAPT* abnormalities can be involved as a disease modifying or risk factor, but TAU pathology appears to be a consequence of the known disease-causing pathology. Hence, TAU might as well have a crucial role in disease pathology and progression of secondary tauopathies, as many pathomechanisms and interconnections are only partially resolved but hint towards TAU involvement. Two questions remain in most cases: 1) Is TAU a bystander, protective (like in NPC and possibly TBI) or a driver of disease (like in AD and PD), and 2) Do the disease-causing genetic abnormalities in secondary tauopathies (such as *LRRK2* in PD or *HTT* in HD) have a direct effect on the physiology of TAU protein, and if so what is the pathomechanistic link? Elucidation of these aspects and further research into the pathomechanistic background of genetic tauopathies will reveal whether TAU is the driver of dementia or the endpoint of a pathological cascade, and whether TAU is a valid therapeutic target for these disorders.

## Data Availability

Not applicable.
